# Episode‐level and clinical characterization of asymptomatic atrial fibrillation events

**DOI:** 10.1111/jce.16423

**Published:** 2024-09-04

**Authors:** Nikhil Ahluwalia, Shubha Majumder, Jodi Koehler, Sean Landman, Shantanu Sarkar, Richard J. Schilling

**Affiliations:** ^1^ St Bartholomew's Hospital, Barts Health NHS Trust London UK; ^2^ Medtronic Inc Minnesota USA

**Keywords:** clinical: implantable devices—atrial fibrillation

## Abstract

**Introduction:**

Not all patients experience debilitating symptoms during Atrial Fibrillation (AF), some are asymptomatic. The reasons for this inter‐ and intrasubject variability is unknown.

**Purpose:**

The study objective was NOAH characterize episode‐level and clinical characteristics associated with symptomatic versus asymptomatic episodes of AF in patients with an implantable cardiac monitor (ICM).

**Methods:**

Patients with an AF episode detected on an ICM between 2007 and 2021 with overlapping clinical data from aggregated Electronic Health Records in the Optum® deidentified data set were included. Symptomatic episodes were labeled in real‐time by the patient. Heart rate (HR) at onset, mean HR, AF Evidence Score (a measure of beat‐to‐beat irregularity), episode duration and Activity Index were evaluated for association with symptom status using multivariable regression modeling.

**Results:**

11 267 patients had AF episodes with clinical data available. The 1776 (15.8%) patients who reported symptomatic AF episodes were younger (67 ± 12 years vs. 71 ± 11 years old, *p* < .001) and had fewer cardiovascular co‐morbidities than patients with asymptomatic AF exclusively. Symptomatic episodes were longer (5.5 [2.4, 14.4] h vs. 3.7 [1.7, 11] h, *p* < .001), had higher mean HR (103 ± 22 bpm vs. 88 ± 22 bpm, *p* < .001) and higher AF evidence scores (98 ± 27 vs. 82 ± 24, *p* < .001). These features were independently associated with symptomatic episodes on multivariable regression analysis and per‐subject analysis in patients who had both symptomatic and asymptomatic episodes.

**Discussion:**

Episode‐level characteristics differed between symptomatic AF episodes versus asymptomatic episodes in patients with ICMs. Symptomatic patients also had less comorbidities. These parameters may be useful in understanding variable symptomatic manifestation and remote stratification of AF episodes.

## Graphical abstract

“Rhythm‐related and clinical differences between symptomatic and asymptomatic Atrial Fibrillation.”

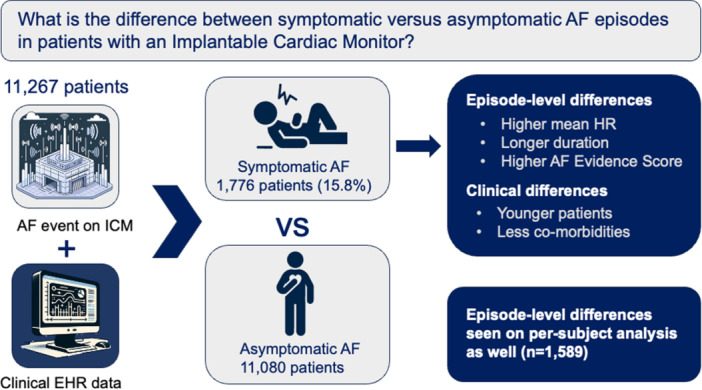

## 1 INTRODUCTION

Atrial Fibrillation (AF) has a worldwide prevalence of 2−4% with a lifetime incidence of one in three for white individuals and one in five for black individuals.^1^ However, the symptomatic impact of AF can vary greatly; both between individuals and within the same person. When AF symptoms occur they are a frequent reason for Emergency Department presentation with two‐thirds of cases resulting in hospitalization.^2^ Traditional AF registries and Randomized ClinicalTrials have predominantly enrolled symptomatic patients who have sought medical attention. The prognostic implications in terms of stroke risk and heart failure hospitalization appears to be independent of symptomatic status.^3^ Asymptomatic AF is under‐represented in traditional AF registries but may a large number of patients based on findings in patients with pre‐existing cardiac implantable electronic devices (CIEDs).^4^, ^5^ Episode‐level evaluation has largely been limited to patients with transvenous CIEDs and wider extrapolation is limited by the inherent disorders of cardiac conduction or structural pathology in this cohort.^6^ The low rate of incidental detection of asymptomatic AF in patients with structurally normal hearts has traditionally prevented comparative assessment of symptomatic versus asymptomatic episodes at scale. Insertable cardiac monitors (ICMs) are increasingly used in patients without known cardiac disease to provide long‐term, continuous heart rhythm surveillance. As the accuracy of AF detection using these devices iteratively improves, reliable cohort‐level evaluation of detected AF events can be performed at scale. This presents a methodology to evaluate episode‐level and clinical characteristics of asymptomatic AF events in patients without transvenous CIEDs.

A comparative evaluation of asymptomatic versus symptomatic AF could inform our understanding of the reasons for inter and intra‐subject variability in reported symptom severity during AF episodes. Patients can temporally label subjective AF symptoms using a compatible handheld activator for long‐term, indelible recording that can be retrospectively correlated with their heart rhythm. Comparing clinical characteristics and episode‐level differences between patients with asymptomatic and symptomatic AF episodes may improve our understanding of the mechanistic drivers of symptomatic manifestation such as differences in heart rate (HR), ventricular rhythm irregularity, or structural features.

This study sought to evaluate and compare differences in episode‐level and clinical characteristics of symptomatic versus asymptomatic AF episodes in a large cohort of patients with structurally normal hearts using linked heart rhythm data derived from ICMs.

## 2 METHODS

### 2.1 Study population and data sources

Patients aged 18 years old or older with AF detected on a Reveal LINQ^TM^ ICM between 2007 and 2021 were identified from a manufacturer's deidentified device data warehouse containing continuous diagnostic monitoring data. (Medtronic Inc. DiscoveryLink). The database was linked to the Optum® deidentified Electronic Health Record (EHR) database and clinical information was derived for identified patients using International Classification of Diseases (ICD)−9/ICD‐10 diagnosis codes and Current Procedural Terminology procedure codes. Patients required 6 months of corresponding EHR data before and after an AF episode onset for study inclusion. Patients with pacemakers, implantable cardiac defibrillation or cardiac resynchronization therapy devices in situ were excluded. The merging of the two deidentified databases was performed by a third party, using a methodology compliant with HIPAA's deidentification standard, such that the overall merged database stays deidentified to all three parties.

This retrospective analysis using deidentified data falls into the category of nonhuman research and is not considered a clinical study, therefore, no Institutional Review Board approval was indicated and was not registered on ClinicalTrials.gov.

### 2.2 Heart rhythm data and episode‐level features

AF detection was based on device manufacturer algorithms that have been previously validated as having an accuracy of >98%.^7^ Briefly, a Lorenz plot of the change in consecutive R‐R interval duration is used to compute the AF Evidence Score; a measure of the degree of irregularity and incoherence of R‐R intervals over the preceding 2‐min period.^8^ If the AF Evidence Score is above a pre‐specified threshold of irregularity, it is labeled as an AF episode. While in an episode, if the AF evidence score falls below the prespecified threshold over 2 min then the episode is terminated. Morphological analysis for P‐wave between consecutive R‐waves as well as patient‐specific device‐learned features of sinus arrhythmia are also incorporated and have been shown to significantly improve AF detection specificity while preserving sensitivity.^9^, ^10^ The most recent version of the AF detection algorithm^10^ had a duration sensitivity and specificity of 98.9% and 99.8% respectively. The algorithm detects 97% of all AF episodes and 85% of all detections are true in patients with known AF. Performance of the algorithm improves with detected AF episode duration with 100% of episodes ≥ 60 min were found to be true in the study in patients with known history of AF.^10^ Extrapolating results from previous studies it is expected that over 98% of all detected AF episodes > 1 h in duration are true AF in the patient cohorts considered in this study.^10^, ^11^

When an AF episode is detected, the electrocardiogram (ECG) waveform, R−R interval time series, and the AF Evidence Score over the first 2 min of the episode are stored in the device. The Activity Index (the number of accelerometer deflections over the first minute following AF episode detection is also stored. Episode duration and mean HR averaged over the entire duration of the AF episode are also calculated and stored. Episode‐level data and the initial ECG waveform are then deidentified and stored in the DiscoveryLink data warehouse. Symptomatic AF episodes were defined as detected AF episodes temporally correlated with patient activation of a handheld symptom activator device within 20 min of AF episode onset. Asymptomatic AF episodes were defined as those detected without any associated activation of the accompanying handheld device. Temporal burden over the first year was analyzed in patients with both symptomatic and asymptomatic AF episodes.

### 2.3 Clinical characteristics and endpoints

Demographic and clinical characteristics of patients were collected, including age, sex, history of coronary artery disease, myocardial infarction, diabetes, and other relevant comorbidities based on the occurrence of ICD‐9 or ICD‐10 codes recorded in the EHR before the onset of the first detected AF episode.

### 2.4 Statistical analysis

Descriptive statistics were used to summarize patient demographics, clinical characteristics, and episode‐level features. Continuous variables were reported as mean ± standard deviation (SD) or median (lower quartile value and upper quartile value) based on the normality of the distribution. Categorical variables were presented as frequencies and percentages. Continuous variables were analyzed using a two‐tailed independent *t*‐test. The Chi‐squared test was used for categorical variables.

Univariable and then multivariable regression modeling was used to evaluate any relationship between selected episode‐level characteristics and symptomatic AF episodes. Selected variables included HR at episode onset (derived from the mean R−R interval duration over the initial 2 min), average HR through the episode, episode duration, activity index, and AF evidence score. A generalized estimating equations model with binomial distribution and exchangeable correlation structure was used to account for multiple AF episodes within a single patient. A per‐subject, paired analysis was also performed in patients with both symptomatic and asymptomatic AF episodes. A Wilcoxon Signed‐Rank test was used for paired data.

Statistical significance was set at a two‐tailed *p* Value of <.05. All statistical analyses were performed using statistical analysis software version X9.4.

## 3 RESULTS

Patient characteristics*:* AF episodes were detected in 11 267 patients during the study period. Episode‐level data was available for all patients. 1776 (15.8%) patients reported at least one symptomatic AF episode whereas 9491 (84.2%) did not report any AF episodes as symptomatic. Indications for ICM implantation in the study population included unexplained syncope (26%), AF management (36%), following cryptogenic stroke (16%) or unexplained palpitations (7%) (Table 1). The baseline clinical characteristics and rates of medications prescribed before the first AF episode are shown in Table 1. The average age was 70 ± 11 years and 56% of patients were male. Patients with symptomatic AF episodes were younger (67 ± 12 years vs. 71 ± 11 years old, *p* < .001) and were more likely to be female (51% vs. 43%, *p* < .001). Patients with symptomatic AF episodes had fewer cardiovascular‐related co‐morbidities including hypertension (63% vs. 74%, *p* < .001), diabetes (19% vs. 29%, *p* < .001), coronary artery disease (31% vs. 39%, *p* < .001) and previous stroke or transient ischaemic attack (20% vs. 33%, *p* < .001). 6924 (61%) patients had a recorded AF diagnosis before the first detected AF episode on the ICM. A further 2296 (20%) patients had an EHR‐confirmed diagnosis of AF made during the follow‐up, thus 9220 (81%) of patients had AF diagnosed by the end of the study. The rates of prescription of beta‐blocker medications and class III antiarrhythmic drugs at baseline were not significantly different between symptomatic versus asymptomatic patients whereas prescription of class I antiarrhythmic drugs was higher in patients with symptomatic AF (16% vs. 10%, *p* < .001). The rates of anticoagulation prescription were similar between both groups (49% vs. 48%, *p* = .40).

**Table 1:** Baseline characteristics of patients included in analysis.

Clinical history	All patients (*n* = 11,267)	Patients with symptomatic AF episodes (*n* = 1776)	Patients without symptomatic AF episode (*n* = 9491)	*p* Value
Mean age (SD)	70 (11)	67 (12)	71 (11)	<.001
Male gender	6299 (56%)	878 (49%)	5421 (57%)	<.001
Hypertension	8111 (72%)	1111 (63%)	7000 (74%)	<.001
Diabetes	3071 (27%)	334 (19%)	2737 (29%)	<.001
CAD	4229 (38%)	542 (31%)	3687 (39%)	<.001
MI	1373 (12%)	151 (9%)	1222 (13%)	<.001
Heart failure	2016 (18%)	204 (11%)	1812 (19%)	<.001
Atrial fibrillation	6924 (61%)	1159 (65%)	5765 (61%)	<.001
Stroke/TIA	3540 (31%)	362 (20%)	3178 (33%)	<.001
CKD	1945 (17%)	186 (10%)	1759 (19%)	<.001
Vascular disease	1517 (13%)	138 (8%)	1379 (15%)	<.001
Medications history				
ACE‐I/ARB	5990 (53%)	791 (45%)	5199 (55%)	<.001
Beta‐blockers	6417 (57%)	1041 (59%)	5376 (57%)	.12
Diuretics	4253 (38%)	544 (31%)	3709 (39%)	<.001
Spironolactone	519 (5%)	63 (4%)	456 (5%)	.02
Sacubitril/valsartan	30 (0.3%)	5 (0.3%)	25 (0.3%)	.89
Vasodilator/nitrate	1997 (18%)	244 (14%)	1753 (18%)	<.001
anti‐arrhythmic drugs				
Class I/III/IV	2994 (27%)	545 (31%)	2449 (26%)	<.001
Class I	1213 (11%)	290 (16%)	923 (10%)	<.001
Class III/IV	2137 (19%)	322 (18%)	1815 (19%)	.33
Anticoagulation	5454 (48%)	876 (49%)	4578 (48%)	.40
Reason for ICM				<.001
AF management	4016 (36%)	738(42%)	3638 (34%)	
Cryptogenic stroke	1837 (16%)	109 (6%)	1728 (18%	
Palpitations	833 (7%)	242 (14%)	591 (6%)	
Suspected AF	1205 (11%)	236 (13%)	969 (10%)	
Syncope	2889 (26%)	378 (21%)	2511 (26%)	
ventricular tachycardia	118 (1%)	14 (1%)	104 (1%)	
Other/unknown	369 (3%)	59 (3%)	310 (3%)	

Abbreviations: AF, Atrial Fibrillation; ICM, Insertable cardiac monitor.

Episode‐level characteristics: 380 625 AF episodes were detected in the included 11 267 patients with a median of 7 (2, 25) episodes per patient. 6190 (1.63%) episodes were marked as symptomatic, with a median of 2^1^, ^4^ episodes marked per symptomatic patient. Stratification by implant indication is shown in Supporting Information S1: Table 3. Symptomatic episodes were longer in duration (5.5 h (2.4, 14.4) vs. 3.7 h (1.7, 11), *p* < .001), had a higher mean HR at onset (105 ± 29 bpm vs. 89 ± 24 bpm, *p* < .001) and higher HR averaged over the duration of the AF episode (103 ± 22 bpm vs. 88 ± 22 bpm, *p* < .001). (Table 2) The AF Evidence Score was significantly greater during symptomatic AF episodes versus asymptomatic episodes (98 ± 27 vs. 82 ± 24, *p* < .001). On multivariable logistic regression analysis, the mean HR over the episode duration, the episode duration and the AF Evidence Score, were independently associated with symptomatic AF (Table 3). The HR during AF had a stronger association with symptomatic AF than the AF Evidence Score.

**Table 2:** Episode‐level characteristics of Atrial Fibrillation (AF) episodes.

	Symptomatic episodes	Asymptomatic episodes	*p* Value
Number of episodes (patients)	6190 (1776)	374 435 (11 080)	NA
AF evidence score (no units)	97.6 ± 26.9	82.1 ± 24.4	<.001
HR (BPM) during entire AF episode	103.0 ± 22.0	87.5 ± 22.0	<.001
Episode duration (h)	5.5 (2.4, 14.4)^a^	3.7 (1.7, 11)^a^	<.001
Activity index (no units)	78.1 ± 60.1	68.6 ± 54.5	<.001
HR (BPM) at onset	105.7 ± 29.3	88.6 ± 27.4	<.001

Abbreviation: HR, heart rate.

^a^ Median (IQR).

**Table 3:** Multivariable regression analysis of AF episode level features and association with symptomatic episodes.

	Univariable (95% CI, *p* Value)	Multivariable (95% CI, *p* Value)
AF evidence score	1.0077 (1.0069‐1.0085, <0.001)	1.0047 (1.0039‐1.0055, <0.001)
Activity	1.0013 (1.0008‐1.0018, <0.001)	ns
Duration (≥6h)	1.54 (1.48‐1.61, <0.001)	1.72 (1.64‐1.81, <0.001)
HR (bpm)	1.0079 (1.0071‐1.0087, <0.001)	ns
HR during AF (bpm)	1.0155 (1.0143‐1.0168, <0.001)	1.0161 (1.0148‐1.0175, <0.001)

Abbreviations: AF, Atrial Fibrillation; HR, heart rate.

Per‐subject analysis: A paired comparison was performed in 1589 patients, who had both symptomatic and asymptomatic episodes (14% of all patients). On per‐subject analysis, the AF evidence score, episode duration and mean HR were significantly greater during symptomatic episodes versus asymptomatic episodes. (Supporting Information S1: Table 1). The prevalence of both symptomatic and asymptomatic AF episodes reduced over the first year since implantation. (Supporting Information S1: Figure 1) The relative reported burden of AF episodes also reduced in these patients.

## 4 DISCUSSION

This analysis is the largest episode‐level characterization of AF episodes using ICM data. ICMs facilitate long‐term, continuous ECG monitoring without transvenous leads. The symptom activator enables patient‐reported, real‐time annotation of concurrent symptomatic episodes to facilitate per‐episode correlation and analysis. In this cohort, symptomatic AF episodes were associated with a greater mean HR and greater irregularity as reflected by a higher AF evidence score on multi‐variate regression modeling. The odds ratio was highly significant but the absolute increase per parameter unit was small. The mean difference on average HR during AF between symptomatic and asymptomatic episodes was 15 bpm. On an individual basis, this would correspond with a 27.1% greater likelihood of symptoms based on the derived odds ratio.

A higher ventricular rate on 12‐lead ECG has been previously reported in symptomatic patients.^12^ A small study has previously reported differences between asymptomatic and symptomatic AF episodes in 25 patients (82 episodes) with ICMs in situ.^13^ HR and SD of successive R−R intervals (SDNN) (a time‐domain measure of HR variability) in the first 2 min of episode onset were manually analyzed. They showed a significantly higher HR and lower SDNN during symptomatic episodes. The pathognomonic features of AF are an increased and irregularly irregular ventricular rate and the loss of active atrial transport. Therefore, demonstrating that these quantitative features may be associated with symptomatic manifestation may account for the variation in reported symptoms and why rate‐control and rhythm‐control therapies may improve symptom severity even in patients who remain in AF. Algorithms based on graphical representations of irregularity such as the Poincare plot or the Lorenz plot are used by wearables and ICMs to discriminate between AF and sinus rhythm.^14^, ^15^, ^16^, ^17^ To our knowledge, this is the first application of the AF evidence score, based on the Lorenz plot, within the AF cohort. Further evaluation of the AF Evidence Score is warranted to evaluate the utility in risk‐stratifying of individual AF episodes.

Patients reporting symptomatic episodes were younger than patients who had asymptomatic episodes only. Symptomatic patients were more likely to have palpitations of unknown cause as their indication for implantation, although the symptom at each activation is unknown. They also had a lower prevalence of hypertension, diabetes and coronary artery disease. The absence of these confounding co‐morbidities may enable patients to attribute symptoms to their AF at onset. Multi‐morbid patients experience a lower health‐related quality of life with AF‐related symptoms such as dyspnoea, fatigue or dizziness due to other co‐morbidities.^18^ Therefore attributing symptoms to AF may be challenging. Our findings are in keeping with findings from the EURObservational research programme‐atrial fibrillation registry, a European observational registry which demonstrated age and cardiovascular co‐morbidities predicted reported asymptomatic status.^19^ However, Individual experience of AF episodes can evolve with symptoms developing or resolving over time. The concordance of our findings on per‐subject evaluation supports the association between episode‐level characteristics and the symptomatic manifestation of a discrete AF episode. This analysis also allowed for controlled comparison for clinical characteristics suggesting mean HR, AF Evidence Score and episode duration are independently associated with symptomatic episodes.

18.2% of patients did not have an ICD‐9/ICD‐10 EHR‐documented AF diagnosis despite having an ICM‐detected AF episode. There was no significant difference in the prevalence of baseline AF diagnoses between patients with symptomatic AF episodes. Randomized controlled trials of patients with CIEDs have reported the clinical implication of device‐detected Atrial High Rate Episodes (AHREs), questioning the clinical role of oral anticoagulation in this setting.^20^, ^21^ These trials predominantly enrolled patients with transvenous devices (1.0% of participants had ICMs in non–vitamin K antagonist oral anticoagulants in patients with atrial high rate episodes 6 and 5.2% in apixaban for the reduction of thrombo‐embolism in ptients With device‐detected subclinical atrial fibrillation). Patients with transvenous CIEDs were excluded from our analysis and a minimum episode duration of 1 h of AF on ICM was used for diagnostic confidence and minimizing the characterization of AHREs.

The ICM population were specifically considered for this study to reduce the potential confounding effect of underlying structural heart disease on episode‐level characteristics. However, all patients indicated ICM implantation with a difference in proportional indication seen between symptomatic and asymptomatic patients. Whether implantation indication affects symptom vigilance and predisposition towards reporting is unknown but may influence reporting behavior.

This study is limited by inherent assumptions of retrospective, observational analyses. The nature of the precipitating symptom causing a patient to activate their ICM was unknown. Symptom labeling using a patient‐held activator is dependent on user compliance and this may lead to an underestimation of symptomatic episodes. Symptomatic episodes that occur during periods when a user may be less likely to access their activator such as at night or during activity may also be under‐represented. Reporting fatigue may also lead to under‐reporting of symptomatic episodes over time. However, labeling behavior has not been studied and should be undertaken to inform the accuracy of this and further device‐based monitoring studies. Using Push Notifications may be a feasible strategy to prompt patients with smartphone‐paired ICMs although this may lead to health anxiety and intrusion.

## 5 CONCLUSION

In patients with an ICM in situ, HR and rhythm parameters during AF episodes and clinical characteristics are significantly different between patients who experience symptomatic AF episodes and those who do not. Episode‐level differences are also associated with symptoms in patients who experience both. This study suggests HR during AF episodes, AF evidence score and episode duration may be associated with the symptomatic manifestation of AF. These parameters may be useful in understanding why the patient experience of AF varies and could be used for the evaluation of symptom‐reduction therapies for AF.

## Supporting information

Supporting information.

## Data Availability

The access to the data that support the findings of this study were licensed from Optum®. Restrictions apply to the availability of these data, which were used under license for this study. Data availability is restricted by the license provided by Optum®.
