# Supplementary material for: Episode‐level and clinical characterization of asymptomatic atrial fibrillation events
Source: J Cardiovasc Electrophysiol. 2024 Sep 4;35(12):2273–9. doi: 10.1111/jce.16423 (PMC11650401; doi:10.1111/jce.16423)

Supplementary Table 1: Characteristics of Atrial Fibrillation episodes in patients who reported both symptomatic and asymptomatic episodes during follow-up

|  | **Symptomatic episodes** | **Asymptomatic episodes** | **P-value** |
| --- | --- | --- | --- |
| **Number of episodes** | 5,899 | 80,182 | NA |
| **AF Evidence Score (no units)** | 97 ± 27 | 84 ± 24 | <0.001 |
| **HR (BPM) During entire AF episode** | 102 ± 23 | 89 ±25 | <0.001 |
| **Episode Duration (hours)** | 6 (2-15) | 4 (2-13) | <0.001 |
| **Activity index (no units)** | 78 ± 60 | 70 ± 57 | <0.001 |
| **HR (BPM) at onset** | 105 ± 29) | 90 ± 28 | <0.001 |

Supplementary Table 2: The International Classification of Diseases (ICD) diagnosis codes used to identify patient co-morbidities from the Optum electronic health record.

| **Disease state** | **ICD-9 or ICD-10 diagnosis codes** |
| --- | --- |
| Hypertension | 401.X, 402.X, 404.X, 403.X, 405.X, I10.X, I11.X, I12.X, I13.X, I15.X |
| Diabetes | 250.X0, 250.X2, E11.X, 250.X1, 250.X3, E10.X |
| Heart failure | 428.X, 402.01, 402.11, 402.91, 404.01, 404.03, 404.11, 404.13, 404.91, 404.93, I50.X, I11.0, I13.0, I13.2 |
| Coronary Artery Disease (CAD) | 410.X, 411.X, 412.X, 413.X, 414.X, I20.X, I21.X, I22.X, I23.X, I25.X |
| Myocardial Infarction (MI) | 410.X, 412.X, I21.X, I22.X, I23.X, I25 |
| Atrial Fibrillation | 427.31, I48.0, I48.1. I48.2. I48.91 |
| Ischemic stroke/ TIA | 433.X, 434.X, 436.X, I63.X, I65.X, I66.X,  435.X, G45.0, G45.1, G45.2, G45.8, G45.9, Z86.73, V12.54 |
| Renal Dysfunction/ CKD | 585.X, 403.X, 404.X, N12.X, N13.X, N18.X |
| Vascular diseases (Myocardial infarction, PAD, aortic plaque) | 440.2X, 443.9, I70.2X, I73.9, 440.0, I70.0 |

Supplementary Table 3: Symptomatic and asymptomatic episode burden stratified by implant indication

| **Implant Indication** | **Symptomatic Episodes (Patients*)** | **Asymptomatic Episodes (Patients*)** |
| --- | --- | --- |
| AF Management | 3273 (832) | 201998 (4349) |
| Cryptogenic Stroke | 314 (113) | 47372 (1855) |
| Palpitations | 817 (230) | 19972 (756) |
| Suspected AF | 821 (210) | 43374 (1104) |
| Syncope | 908 (365) | 49023 (2791) |
| Ventricular Tachycardia | 38 (12) | 5297 (108) |
| Other | 17 (1) | 385 (10) |

*More than one indication can be recorded per implant, therefore cumulative sum of patient number may be greater than total cohort size

Supplementary Figure 1: Stacked histogram to show the proportion of participants with AF episodes recorded over time


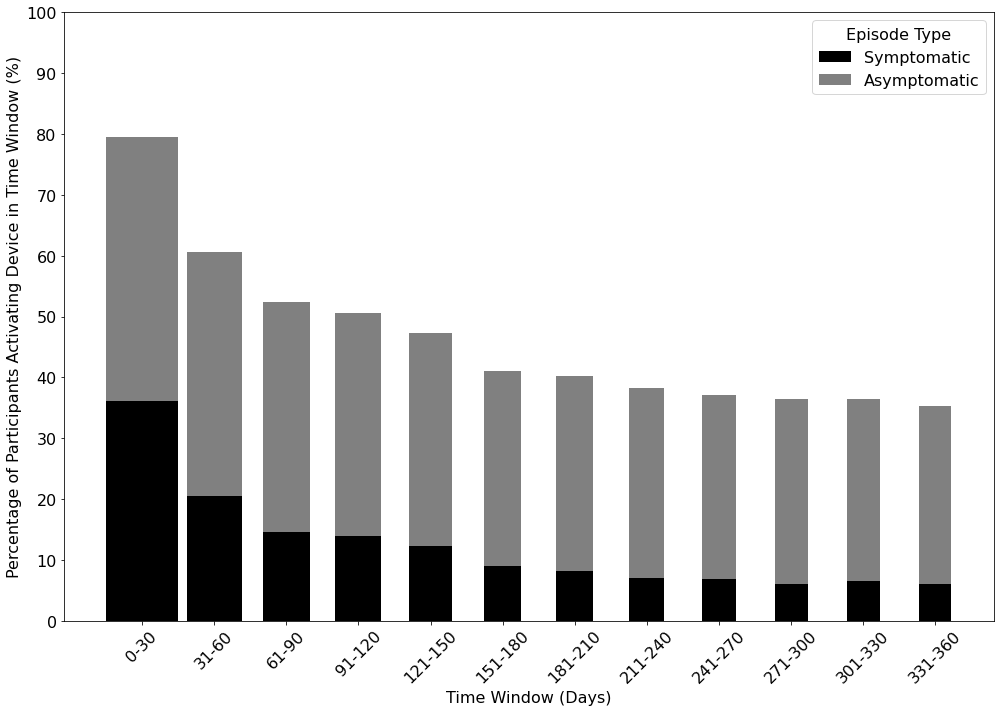

Supplement: Supplementary file 1 — Supporting information. [file JCE-35-2273-s001.docx]
